# Characterization of a novel nanobiomaterial fabricated from HA, TiO_2_ and Al_2_O_3_ powders: an in vitro study

**DOI:** 10.1007/s40204-014-0025-8

**Published:** 2014-06-14

**Authors:** Mahboobeh Mahmoodi, Peyman Mahmoodi Hashemi, Rana Imani

**Affiliations:** 1 Department of Materials and Mechanic, Yazd Branch, Islamic Azad University, Yazd, Iran; 2 Department of Biomedical Engineering, College of Engineering and Technical, Yazd Science and Research Branch, Islamic Azad University, Yazd, Iran; 3 Biomaterial Group, Faculty of Biomedical Engineering, Amirkabir University of Technology, Tehran, Iran

**Keywords:** Nanobiomaterial, Sintering, Hydroxyapatite, Titania, Bioactivity, Cells proliferate

## Abstract

For the purposes of this study, hydroxyapatite (HA)–Al_2_O_3_–TiO_2_ nanobiomaterial with significant surface properties and biocompatibility capable of forming surface apatite was fabricated by cold-press and sintering method. Samples were examined for hardness and porosity. The results showed that in terms of hardness and porosity, sample A (50 wt% TiO_2_–30 wt% HA–20 wt% Al_2_O_3_) was superior to sample B (30 wt% TiO_2_–50 wt% HA–20 wt% Al_2_O_3_), and also the density of nanobiomaterial was close to natural bone density. Bioactivity of the samples in a simulated body fluid (SBF) was investigated. Then, after immersing the samples in SBF solution for a period of 7 days, sample B exhibited greater ability to form calcium phosphate compounds on the surface as compared to sample A. In addition, in vitro studies showed that MG-67 osteoblast-like cells attached and spread on the samples surface. The results showed that cells proliferated in greater numbers on the sample B as compared to the sample A. Finally, X-ray diffraction, scanning electron microscopy, and energy-dispersive X-ray analysis were performed to identify phases, study microstructure, and determine percentage of elements, respectively. The results revealed that considering their different properties, both nanobiomaterials can be used in medical applications.

## Introduction

Development of new biomaterials for medical applications is of primary concern to researchers. Orthopedics is one of those sciences that typically requires such materials for healing and replacing missing parts (Aubry et al. 2009; Dorozhkin 2010; Murugan and Ramakrishna 2005; Seal et al. 2001; Bellucci et al. 2011; Ashokkumar and Sangeetha 2013). Today, various synthetic materials including composites as a bone substitute have been developed to overcome problems associated with bone defect repair (Lee and Shin 2007; Sivakumar and Panduranga Rao 2002; Uemura et al. 2003; Yoneda et al. 2005; Nezafati et al. 2013). Bone is a natural composite whose mineral part is formed by hydroxyapatite (HA) and is reinforced by collagen (Olszta et al. 2007; Sun et al. 2011; Ngiam et al. 2009; Nandakumar et al. 2013; Andiappan et al. 2013). In many fractures and bone defects, substitute materials or fillers are required to repair bone tissue. A material with chemical and mechanical properties as bone cannot singularly be found, thus, biomedical composites are often designed to provide good biocompatibility and mechanical behavior (Chen et al. 2004; Scholz et al. 2011; Cao et al. 2011; Rath et al. 2012).

HA is a biocompatible ceramic used in orthopedic and dental implant applications with very similar chemical compositions to the mineral part of bone and tooth and can establish a good bond with bone tissue. However, application of HA due to low toughness and weak bending strength is limited under loading conditions (Rezwan et al. 2006; Swetha et al. 2010; Zhou and Lee 2011; Balani et al. 2007; Abdal-hay et al. 2013). Therefore, it is used as filler in small bone defects and as a coating on metal implants such as titanium (Bai et al. 2010a; Sadat-Shojai et al. 2010; Sato et al. 2006). Recent studies have reported improved ossification process and implant fixation through various methods including plasma spray and laser (Roy et al. 2011; Topić et al. 2006; Khosroshahi et al. 2009, 2007).

Today, production of HA nanocomposite has made design of new materials with bone-like structures possible. Such new materials create high chemical homogeneity of coatings and allow production of dense composites at low sinter temperatures (Aminzare et al. 2013; Andronescu 1993). Also, bioactive composites can be attached to bone with formation of HA layer on the surface. According to recent reports, HA-based composites with reinforcements such as CNT, TiO_2_, ZrO_2_, Al_2_O_3_ to improve mechanical properties have attracted the interest of researchers (Bai et al. 2010b; Kalmodia et al. 2010; Kratschmer and Aneziris 2011; Wen et al. 2007; Sopyan et al. 2012).

The Al_2_O_3_ bio-ceramic (alumina) increases fracture toughness and wear resistance, while maintaining biocompatibility due to its inert nature. It is also able to considerably increase thermal resistance of composites (Kalmodia et al. 2010).

Titania (TiO_2_) is another common reinforcement for composites. While being biocompatible, antibacterial, and photo-catalyst, the presence of this bio-ceramic enhances corrosion resistance of implants (Cho et al. 2008). Also, it is able to absorb H_2_O and form titanium hydroxide (Ti–OH) groups on the surface, which is a factor in formation of apatite in simulated body fluid (SBF) (Beherei et al. 2009). There are reports of fabrication of Al_2_O_3_/TiO_2_ (Habibpanah et al. 2011), HA/TiO_2_ (Enayati-Jazi et al. 2012), HA/Al_2_O_3_ (Viswanath and Ravishankar 2006) composites for medical applications. However, there are no reports of fabrication HA–TiO_2_–Al_2_O_3_ nanobiomaterial by cold-press and sintering method. Thus, in this study, two HA–TiO_2_–Al_2_O_3_ nanobiomaterials with different weight percentage of ingredients fabricated by cold press and sintering method. Finally, the biocompatibility and surface properties of both nanobiomaterials compared for medical applications.

## Materials and methods

### Preparation of nanobiomaterials

To synthesize the nanobiomaterial, a 2-cm wide cylindrical steel mold was prepared to cold press the raw materials. Then, rutile-TiO_2_ (20 nm), alpha-Al_2_O_3_ (80 nm), and HA (1 µm) powders as biomaterial ingredients, and sodium silicate for improving adhesion between particles were purchased from the Merck Company. Two samples with different mounts of powders were made (Table 1). In sample A, titania (50 wt%), HA (30 wt%) and alumina (20 wt%) were mixed. HA and alumina particles are as reinforcement materials in sample A. In sample B, HA (50 wt%), titania (30 wt%) and alumina (20 wt%) were mixed in which titania and alumina nanoparticles are as reinforcement materials.

**Table 1:** The weight percentages of materials of nanobiomaterials

Sample	Al_2_O_3_ (wt%)	TiO_2_ (wt%)	HA (wt%)
A	20	50	30
B	20	30	50

To synthesis nanobiomaterial, different weight percentages of the powders (Table 1) were mixed with sodium silicate and for more homogeneity, powder mixtures were milled in a polymeric ball mill at the speed of 400 rpm. Then 4 g of the mixture was poured into the steel mold and compressed with a uniaxial cold press at a pressure of 150 kg/cm^2^ (14.7 MPa). Next, the samples were dried in an oven at 150 °C for 2 h. To increase the strength, samples were sintered at 1,000 °C for 40 min, and allowed to cool down at the oven temperature. The hardness of samples was measured and compared before and after sintering.

### Morphology and microstructure

The surface morphology and elemental map of the samples were examined with scanning electron microscope (SEM, VEGA II, and Tescan, USA). Images were taken of the horizontal cross-section of samples before and after immersion in SBF, at different magnifications. To analyze percentage of elements in samples was used energy-dispersive X-ray analysis (EDAX). Also, bulk density and apparent porosity of sintered samples were measured using Archimedes’ method by immersing samples in water (Wan et al. 2008).

### Surface hardness

To measure surface hardness of the samples in Vickers, a square-based pyramid with 136° angle between the opposite faces was used as punch. The Vickers hardness number (VHN) is defined as load divided by area of depression. In practice, this area is calculated from microscopic quantities of lengths and diameters using Eq. (1) (Chen et al. 2011).

1 $$\text{VHN}=\frac{1.854P}{L^{2}}$$

Where, *P* is applied load in grams, *L* is average diagonal size of indentation in mm and ϴ is angle between opposite faces of diamond indenter. To find the surface hardness, samples were placed under the Vickers punch, and a load of 200 gf with dwell time of 10 s was applied.

### In vitro bioactivity studies

To evaluate the in vitro bioactive behavior of the samples, the SBF solution was prepared in accordance with Kokubo Instruction (Kokubo et al. 1999). SBF compounds similar to human blood plasma are presented in Table 2. After sintering the samples, they were immersed in 60 ml of SBF and then incubated for a period of 7 days at body temperature (37 °C) with 98 % humidity and 5 % CO_2_. Each day, the SBF solution on the samples was replaced with a fresh solution. At the end of the experiment, percentage of elements and surface morphology of samples were evaluated by EDXA analysis and SEM microscope.

**Table 2:** SBF and human blood plasma ion concentrations (mmol/L)

Ion	Na^+^	K^+^	Ca^2+^	Mg^2+^	HCO^3−^	Cl^−^	HPO_4_^2−^	SO_4_^2−^
SBF	142	0.5	2.5	1.5	4.2	147.8	1	0.5
Blood plasma	142	0.5	2.5	1.5	27	103	1	0.5

### X-ray diffraction analysis

The X-ray diffraction pattern test (XRD) (Philips, X’ Pert Pro) was performed to identify the phases formed in samples, and to examine their crystallization rates. The phases in samples were evaluated by means of the Panalytical Software X’ Pert High score Plus and database PDF-2 (David et al. 2007).

### In vitro cell culture

The in vitro cytotoxicity behavior of samples was evaluated and compared for a minimum incubation period of 3 days using the human MG-67 osteoblast-like cells (osteosarcoma cell line, ATCC, Invitrogen, Carlsbad CA USA). All samples were sterilized by autoclaving at 121 °C for 20 min prior to the cell culture experiment. Following this, cells were seeded onto surface of samples and a negative control (i.e., MG-67cells only in the cell culture medium) then placed in a 24-well tissue culture polystyrene (TPS). The initial cell density was 3.0 × 10^4^ cells wall^−1^. One milliliter of Dulbecco’s modified Eagle’s medium (DMEM) enriched with 10 % fetal bovine serum was added to each well. Based on ATCC’s MG-67cell culture protocol, cultures were maintained at 34 °C under an atmosphere of 5 % CO_2_ and 95 % air for up to 3 days for cell attachment. The culture media were changed every alternate day (Kim et al. 2004).

#### Cell morphology

Cell morphology was assessed after 3 days of incubation period by SEM. Cultured samples for SEM observation were rinsed with 0.1 M phosphate-buffered saline (PBS) and fixed with 2 % paraformaldehyde/2 % glutaraldehyde in 0.1 M cacodylate buffer overnight at 4 °C. Following this, post-fixation was performed for each sample with 2 % osmium tetroxide (OsO_4_) for 2 h at room temperature. Fixed samples were then dehydrated in an ethanol series (60, 70, 80, 90 and 100 % three times), followed by a hexamethyldisilane (HMDS) drying procedure. Dried samples were then mounted on aluminum stubs, gold coated and observed by SEM (Kim et al. 2004).

#### Cell proliferation using the MTT assay

The proliferation of viable MG-67cells attached on A and B sample surfaces was assessed by the MTT assay (Sigma, MO, USA) after 3 days of incubation. A 5 mg ml^−1^ solution of MTT [3-(4,5-dimethylthiazol-2-yl)-2,5-diphenyl tetrazolium] was prepared by dissolving MTT in PBS and filter sterilizing it. MTT solution was diluted (100 µl into 900 µl) with DMEM culture medium enriched with 10 % fetal bovine and added to each sample to form formazan through the action of mitochondrial dehydrogenases. After 2 h incubation at 34 °C, samples were transferred to a new 24-well plate and 1 ml of solubilization solution made up of 10 % Triton X-100, 0.1 N HCl and isopropanol was added to dissolve the formazan crystals. Then 100 µl of the resulting supernatant was transferred into a 96-well plate and three data points were obtained from each sample. The optical density of the solution in each well was measured at a wavelength of 570 nm using a microplate reader (BioTek, Elx808, USA) (Huang et al. 2011).

## Results and discussion

### Morphology and apparent porosity

**Fig. 1:**
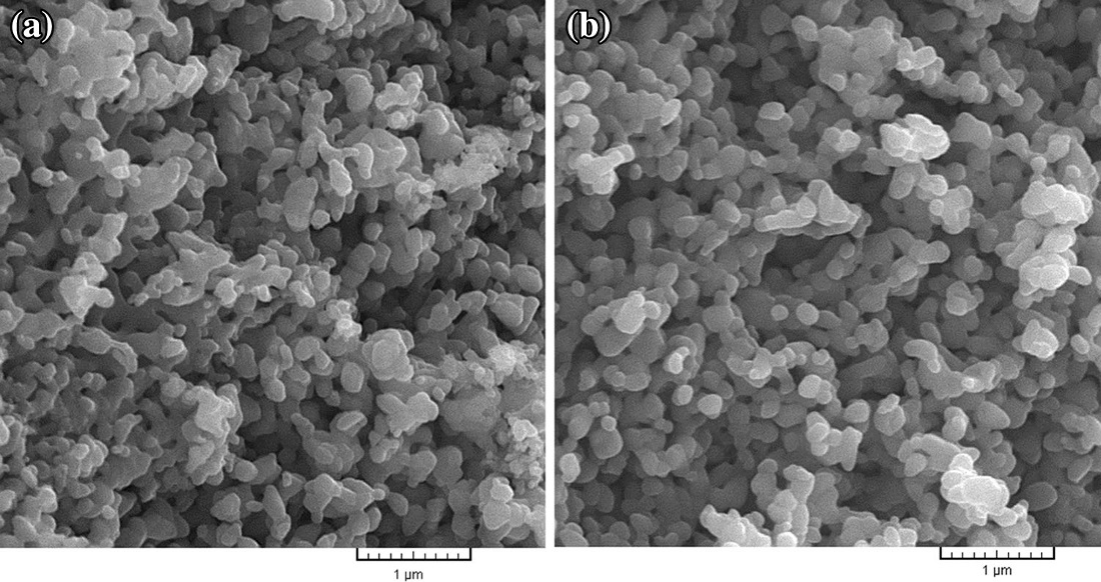
SEM images **a** sample A, **b** sample B before immersion in SBF

Microstructure and morphology of the surface of samples A and B were assessed by observation of SEM images. The SEM images (Fig. 1) show that the surface of both types of samples is fully sintered, which is indicative of well-bonded particles and correct density and porosity of the samples. However, the particle size varies after sintering and average of pore size in the surface of samples A and B is between 100 and 300 nm. The difference in thermal expansion coefficient of the composite ingredients during heating and cooling can lead to formation of micro-cracks (Kwok et al. 2009). In this study, to prevent formation of these cracks, samples were sintered at 1,000 °C for 40 min. As can be seen in Fig. 1, there are no cracks on the surface of the samples. Table 3 shows the chemical composition of nanobiomaterials.

**Table 3:** EDAX results of nanobiomaterials before immersion in SBF

Sample	Ca (%)	Ti (%)	O_2_ (%)	P (%)	Al (%)
A	7.76	26.53	53.77	4.45	7.49
B	13.43	18.91	53.38	7.1	7.18

**Fig. 2:**
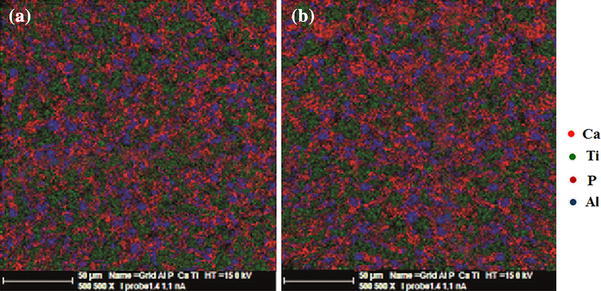
Elemental map **a** sample A, **b** sample B

The result of samples combination can also be visualized giving a map of elements of particles under the microscope. A homogeneous distribution of nanobiomaterial ingredients (P, Ca, Ti, Al) in structure of all samples is shown in Fig. 2. The percentage porosity and density of the samples are presented in Table 4. Sample B had a better particle size distribution, smoother surface, and less porosity (31.3 %) than sample A (41.99 %). An increase in weight percentage of the nano-sized TiO_2_ compared to the micron-sized HA in nanobiomaterial A amplifies the likelihood of a porous surface (Harle et al. 2006). Consequently, with increased percentage of porosity in sample A, vacant space is provided for growth and nutrition of bone cells. But, with reduced HA, the bioactive and osteoconductive properties of this implant were decreased. Samples A and B have densities of 1.994 and 2.016 g/cm^3^, respectively, which are very close to the bone density (1.85 g/cm^3^). Of course, density of sample A compared to sample B has decreased due to the use of nano-sized TiO_2_. Use of HA particles as matrix and addition of titania (30 wt%) and alumina (20 wt%) nanoparticles as reinforcing phase in sample B have improved bioactivity of implant in areas where better osteogenesis and stability of implant and more adhesion of nanobiomaterial to the bone are needed.

**Table 4:** Percentage porosity and density of nanobiomaterials

Sample	Porosity (V %)	Density (g/cm^3^)
A	41.99	1.994
B	31.30	2.016

### X-ray diffraction pattern

**Fig. 3:**
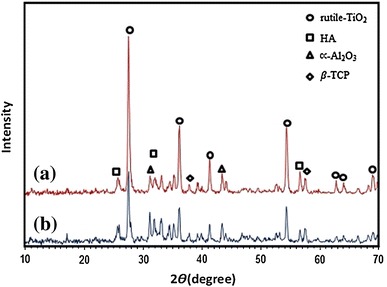
X-ray diffraction pattern of nanobiomaterial **a** sample A, **b** sample B before immersion in SBF

The X-ray diffraction patterns of A and B samples before immersion in SBF solution can be observed in Fig. 3. Peaks associated with HA are detected with intensity near 2ϴ = 26° and 2ϴ = 32° in (201) and (211) planes, respectively. Also, β-TCP peak can be observed around 2ϴ = 31.5° in (221) plane, which is very close to HA peak. The TiO_2_ peak with a high intensity is observed near 2ϴ = 36° in (110) crystal plane. Another reinforcement in nanobiomaterial is alumina that can be seen near 2ϴ = 38° and 2ϴ = 58° in (110) and (116) planes. But Al_2_O_3_ has a bigger peak at 2ϴ = 35.5° that is integrated with Titania peak at 2ϴ = 36°. The diffraction pattern peak intensity of the TiO_2_ is higher in sample A. It can be seen from the results that after sintering nanobiomaterial for 40 min at 1,000 °C, the nanobiomaterial ingredients maintained their nature, and β-TCP peak with a low intensity is observed in the samples. The intensity of β-TCP peak was higher in sample B due to higher percentage of HA. Also, there was not any peak around 2ϴ = 30° thus, calcium aluminate phase was not observed.

Although HA is constant and stable in the body, the presence of secondary phases causes it to dissolve, which consequently leads to degeneration of the implant in body. Thus, increased sintering temperature followed by higher crystallization is necessary for longer life of the implant. Furthermore, sintering temperatures higher than 800 °C lead to decomposition of HA to β-TCP and α-TCP, whose presence in the sample reduces biocompatibility of the implant. Also, when samples are placed in biological environments like blood plasma, β-TCP becomes unstable and gradually degenerates. Thus, the presence of this phase could be considered responsible for the composite strength loss after exposure in SBF solution (Kwok et al. 2009). However, in this study, due to sintering temperature (1,000 °C), the intensity of β-TCP peak was low in the structure of both samples, but this weak peak cannot much affect biocompatibility of the samples. During high temperature processes (1,200–1,400 °C) in synthesis of nanomaterial, due to increased surface and reaction area, new phases such as calcium aluminates are created that reduce mechanical properties of the material (Viswanath and Ravishankar 2006). In this study, to prevent formation of secondary phases such as calcium aluminate phase, sintering operations were conducted at 1,000 °C.

### In vitro bioactivity evaluation

**Fig. 4:**
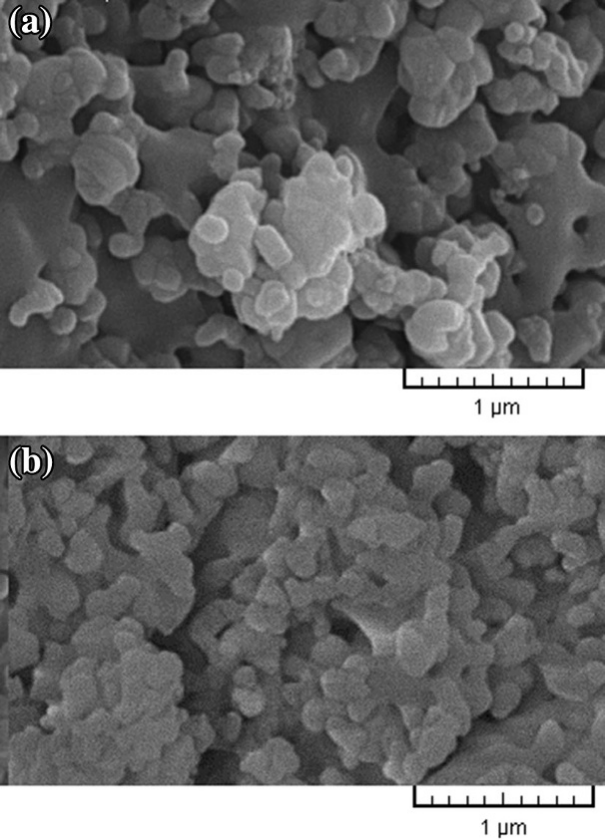
SEM images **a** sample A, **b** sample B after immersion in SBF

The in vitro bioactive behavior and formation of calcium phosphate phase on the surface of nanobiomaterial in SBF solution were evaluated by SEM images and EDXA analysis. Figure 4 shows SEM images of A and B samples after immersion in SBF solution, and formation of apatite can be observed on both of surfaces. But, there is a considerable difference in ability to form apatite in sample B compared to A. The higher percentage of HA and TiO_2_ nanoparticles present in sample B is due to full coverage of its surface by apatite, while in sample A, apatite is scattered in different areas of surface. Researchers have recently reported (Kong et al. 2006) that the increase in HA in composites encourages the formation of apatite on the surface of samples in SBF solution.

The biological activity of bioceramics is due to their ability to promote the formation of HA in physiological environments (SBF) (Fujibayashi et al. 2003; Rámila and Vallet-Regi´ 2001). In this study, formation of apatite and calcium phosphates on ceramic nanobiomaterial was assessed and the relationship between apatite formation and bioactivity of nanobiomaterial was identified. It can be seen from EDAX analysis (Table 5) that calcium and phosphorus levels significantly increased after 7 days immersion of samples in SBF compared to their levels before immersion (Table 3). Also, the percentages of Ti, O_2_ and Al decreased with formation of an apatite-like material on the nanobiomaterial surface. The ratio of calcium to phosphorus is almost 1.7 and this ratio did not change much in the samples after immersion in SBF. Calcium ion concentration is controlled by the formation of apatite layer in SBF and its release from the samples (Martínez et al. 2000).

**Table 5:** EDXA results of nanobiomaterials after 7 days immersion in SBF

Sample	Ca (%)	Ti (%)	O_2_ (%)	P (%)	Al (%)
A	18.26	20.75	47.61	9.74	3.64
B	25.6	8.33	49.26	14.62	2.19

### Surface hardness and reasons for fabrication of nanobiomaterial with 3 materials

Table 6 presents the results of the surface hardness before and after sintering operation. The surface hardness of both A and B samples nearly doubled after sintering. Obviously, this increase in surface hardness was due to sintering. However, non-homogeneous distribution of powders in nanobiomaterial structures could cause a reduction in hardness and strength of the samples. The surface hardness of sample B before and after sintering was almost half that of sample A, and since the amount of Al_2_O_3_ was constant in both of samples; this may have been due to reduction in the amount of TiO_2_ in sample B. Another reason for reduced hardness in sample B compared to sample A could be higher formation of beta-tri-calcium phosphate phase in sample B because of increasing in the amount of HA.

**Table 6:** The surface hardness of nanobiomaterials before and after sintering operation

Sample	Hardness before sintering (GPa)	Hardness after sintering (GPa)
A	3.09	7.01
B	1.54	4.25

HA is widely used as a biomaterial in medical applications, and alone can amplify bioactivity and tendency to absorb biological materials like protein but, when HA coats on titanium substrate, mechanical properties (fracture toughness) and adhesion strength are weak. Research shows that the presence of TiO_2_ alongside HA in composite and HA coating on Ti surface causes increased adhesion strength and corrosion resistance (Wen et al. 2007). In addition, it can elevate surface hardness without compromising formation of apatite. The presence of Al_2_O_3_ in the sample does not affect calcinations process (Li et al. 1995). Besides, addition of 20 % Al_2_O_3_ almost doubled fracture toughness and HA strength (Viswanath and Ravishankar 2006). As reinforcement in composites, TiO_2_ is able to absorb H_2_O and form Ti–OH groups on the surface, which eventually leads to the formation of apatite in SBF solution and enhances bioactivity of samples (Madhan Kumar and Rajendran 2013). Therefore, this reinforcement material has an important role in adhesion of implant to the bone (Beherei et al. 2009).Particle size and distribution of ingredients in composites affect their strengths. Thus, in this study, 3-phase ceramic HA–TiO_2_–Al_2_O_3_ was fabricated.

### In vitro behavior of nanobiomaterials

**Fig. 5:**
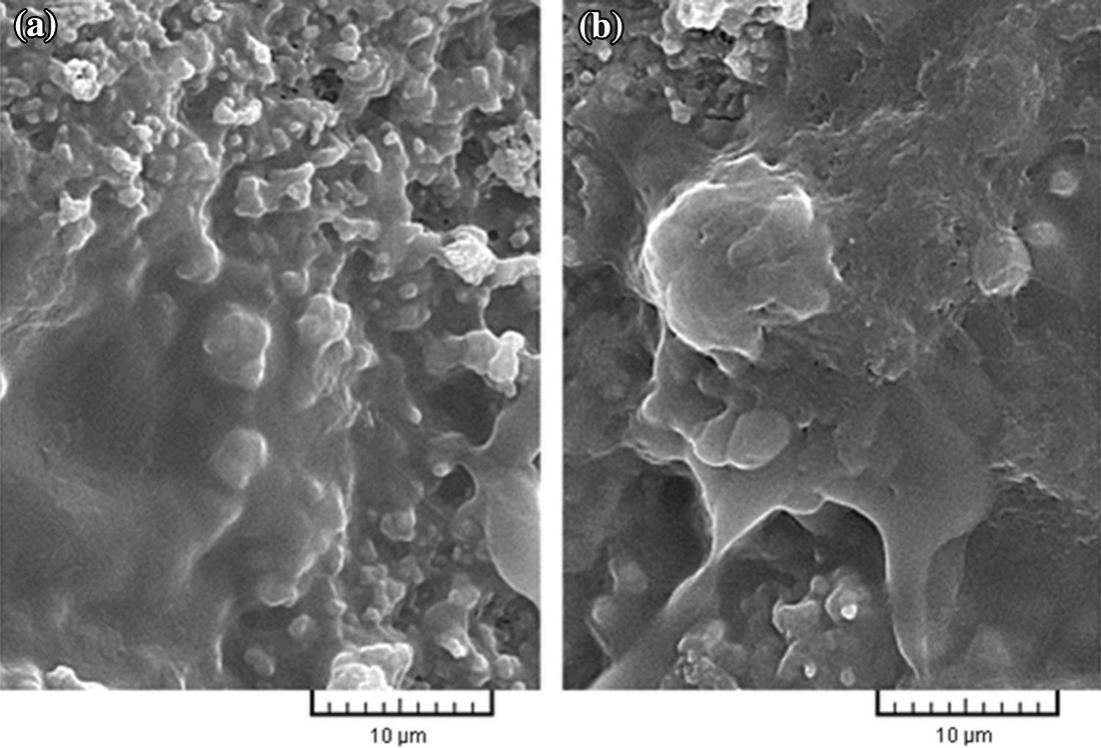
SEM micrographs illustrating MG-67cell morphology after 3 days of culture on **a** sample A, and **b** sample B

**Fig. 6:**
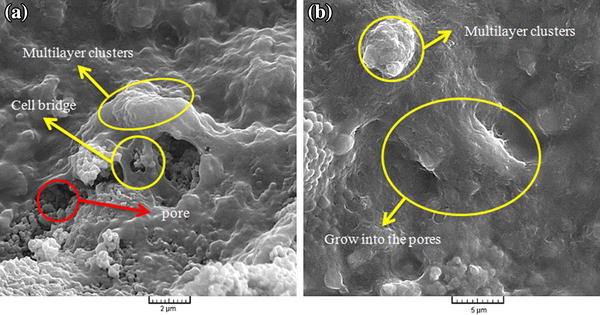
high-magnification SEM micrographs illustrating MG-67cell morphology after 3 days of culture on **a** sample A, and **b** sample B

SEM observation revealed the MG-67cell attachment, growth and spreading on different sample surfaces. Figure 5 shows the morphologies of the cells on samples after 3 days of culture. Cells were flattened and well spread across the A and B sample surfaces after 3 days. On both the A and B sample surfaces, cells were seen to adhere to each other with cellular micro-extensions (e.g., filopodia), and were connected to the substrate in addition to neighboring cells. Cells were also found to have migrated into the pores and were observed to bridge them. However, on the A surface, relatively fewer cells were observed (Fig. 6a), but on the B sample, as is shown in the high-magnification SEM image in Fig. 6b, cells were observed to grow into the pores. On the B surface cell numbers were increased, and the entire sample surface was covered with cells having numerous filopodia extensions attached to the surface irregularities, as shown in Fig. 5b.

After 3 days of culture, cells appeared to be more elongated and confluent on B, as shown in Fig. 6b. Evidently, granules were deposited on the cell surfaces because of extracellular matrix (ECM) mineralization. In contrast, on sample A, cells never reached complete confluence over the entire sample surface because of the higher pore volume. As shown in Fig. 6, on both of samples, cells formed multilayer clusters (i.e., Cells appeared cuboidal and had a three-dimensional morphology with more filopodia) on the smooth surface area. Also, nano-scale grains and high volume fraction of grain boundaries in HA nanomaterial can increase adhesion of osteoblasts, cell proliferation, and mineralization of these composites (Zhang and Kwok 2011).

**Fig. 7:**
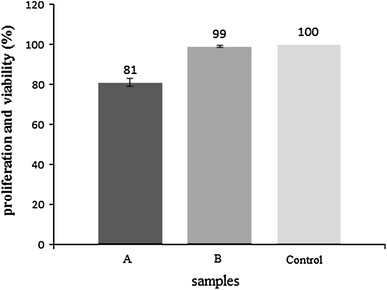
MTT assay of cells on A and B samples after 3 days of incubation

The MTT assay was used to quantitatively determine the proliferation of viable MG-67cells on the A and B sample surfaces. Figure 7 shows a comparison of viable cell densities for samples with negative control after 3 days of culture. For culture durations, cells proliferated in greater numbers on the sample B compared to the sample A. It was observed that, cells proliferated most rapidly on the samples with the highest porosity, i.e., on B in comparison to A sample. Therefore, the results revealed that both nanobiomaterials can be used in different medical applications.

## Conclusions

In this study, nanobiomaterial HA–TiO_2_–Al_2_O_3_ was fabricated with cold-press and sintering method, and with desired surface properties. The bioactivity and formation of calcium phosphate phase on the surface nanobiomaterial in SBF solution were investigated. On the surface of sample B with higher weight percentage of HA, more apatite and calcium phosphate phases were formed as compared to sample A. Also, density of the nanobiomaterial was near that of natural bone. The surface hardness increased after sintering in both samples, while surface hardness of sample A with higher weight percentage of TiO_2_ before and after sintering was more than that in sample B. Creation of a surface without micro-cracks, reduction in creation of β-TCP phase, and prevention of formation of α-Ca_3_ (PO_4_)_2_ phase were other results achieved in this study. In addition, on both of the A and B sample surfaces, cells were seen to adhere to each other with cellular micro-extensions but on the B surface cell numbers were increased, and the entire sample surface was covered with cells. Therefore, given the different properties of the nanobiomaterial fabricated, both can be used in different orthopedics and dental implant applications. Sample B can be used to improve implant bioactivity in areas that need improved osteogenesis, fixation of bone implant and bone filling, and increased adhesion of nanobiomaterial to the bone. Meanwhile, sample A can be used in areas that need coating to increase biocompatibility, increased adhesion, and implant strength.
